# Development of a New Bioadhesive Gel in the Prevention of Bacterial Biofilms on Dental Implants: An In Vitro Study

**DOI:** 10.1002/cre2.70422

**Published:** 2026-09-27

**Authors:** María Baus‐Domínguez, Paula Hermida‐Cabrera, Javier Pascual, Fernando Vivancos‐Cuadras, María‐Ángeles Serrera‐Figallo, José‐Luis Gutiérrez‐Pérez, Daniel Torres‐Lagares

**Affiliations:** ^1^ Department of Stomatology, Faculty of Dentistry University of Seville Seville Spain; ^2^ Faculty of Health Sciences Universidad Europea de Valencia Valencia Spain; ^3^ LACER Medical Department Barcelona Spain

**Keywords:** bioadhesive gel, biofilm, clinical research, implant surface

## Abstract

**Objectives:**

Peri‐implantitis is a reality that clinicians face in their daily practice. Current evidence discusses the applicability of bacteriostatic or bactericidal methods in order to minimize the colonization of bacteria, which entails controversy. In this scenario there arises a need to develop surfaces that minimize bacteria's appetite for them.

**Methods:**

An in vitro microbiological study was conducted to assess the effect of a chlorhexidine‐based bioadhesive gel on the growth and adhesion of a simplified peri‐implantitis–associated microbial consortium. Grade IV titanium disks with three surface types (machined, sandblasted, and ContacTi) were incubated with the consortium under anaerobic conditions for 10 days, with and without gel treatment. Microbial viability was evaluated by colony‐forming unit (CFU) counts, and biofilm structure was analyzed by scanning electron microscopy. Microbial community composition was assessed using 16S rRNA gene amplicon sequencing.

**Results:**

After 10 days, gel‐treated implants showed complete inhibition of cultivable microorganisms, with CFU values reduced below the detection limit on all surfaces, compared with untreated implants (*p* < 0.05). In untreated samples, ContacTi surfaces exhibited higher bacterial viability than machined and sandblasted surfaces. Metataxonomic analysis revealed a marked reduction in peri‐implant pathogens, particularly *Porphyromonas gingivalis*, whose relative abundance decreased from approximately 20% in the initial inoculum to ~1% after incubation.

**Conclusions:**

The bioadhesive gel demonstrated a quantitatively significant antimicrobial effect, preventing biofilm formation on all tested implant surfaces after 10 days.

## Summary

**Clinical relevance:** Peri‐implantitis remains a major clinical challenge in implant dentistry, defined by bleeding on probing, probing depths ≥ 6 mm, and marginal bone loss exceeding 3 mm. Its pathogenesis is strongly associated with microbial biofilm formation on implant surfaces, with surface topography playing a critical role in microbial adhesion and colonization. This study addresses the pressing need to better understand the interactions between implant surface characteristics—specifically sandblasted, machined, and ContacTi surfaces—and complex polymicrobial communities. It further evaluates the potential of bioadhesive gels containing biocidal agents to prevent or disrupt biofilm formation, in light of the current variability and limitations in the effectiveness of adjunctive antimicrobial strategies.

**Principal findings:** Application of the bioadhesive gel resulted in complete inhibition of bacterial growth and biofilm formation across all implant surfaces after 10 days of exposure. In untreated samples, biofilm development and microbial viability varied according to surface type, with ContacTi surfaces supporting higher bacterial survival. Notably, the gel exerted a more substantial influence on microbial community composition than the implant surface itself, reshaping the relative abundance of key taxa. These findings confirm that biofilm formation is possible even on minimally rough surfaces, underscoring the necessity for adjunctive antimicrobial approaches beyond surface design alone.

**Practical implications:** This study supports the use of bioadhesive gels as a promising adjunctive strategy for biofilm prevention in implant therapy. Their application may enhance peri‐implant disease management, particularly in cases involving surfaces with increased susceptibility to microbial colonization. The in vitro evidence presented here provides a solid foundation for future in vivo and clinical investigations, aimed at integrating such antimicrobial formulations into routine implant maintenance protocols without compromising osseointegration.

## 1 Introduction

Using implants to replace missing teeth is an effective solution in most cases, with a cumulative survival rate of 94% over the past 15 years (French et al. 2021). Unlike other fixed prosthetic solutions, dental implants do not require the sacrifice of adjacent teeth, maintaining their integrity (Elani et al. 2018). Due to this increase in the use of dental implants, implant‐related pathologies are also on the rise. Treating periodontitis in its various stages is no longer the only challenge for the clinician; peri‐implantitis and mucositis are also becoming more common in dental practices, and the practitioner must diagnose and treat them early. Still, peri‐implantitis and mucositis are becoming more common in dental practices, and the practitioner must diagnose and treat them early (Pavel et al. 2012).

Peri‐implantitis is a pathological entity associated with biofilm formation on the surface of dental implants. It is characterized by chronicity and marginal bone loss, which differentiates it from mucositis. Its occurrence poses a risk to the longevity of implants (Carcuac et al. 2016).

Biofilm has been established as the etiological agent of peri‐implantitis; however, recent studies have identified other risk factors that predispose to the development of the pathology, such as lack of maintenance, a previous history of periodontitis, deleterious patient habits, poor spatial location of the implants and, therefore, of the prosthetic rehabilitation, over‐contoured prostheses, and lack of keratinized gingiva (Schwarz et al. 2018).

In this context, numerous surgical and non‐surgical therapies have emerged to alleviate the problem, ranging from mechanical debridement and maintenance to resective surgery and even regenerative procedures. They all have a common focus: reducing or eliminating the bacterial load and achieving a healthy state of the peri‐implant tissues and implant success (Ramanauskaite et al. 2021).

Due to the bacterial nature of peri‐implant pathology, the literature has also analyzed the use of antibiotics at a systemic and localized level, which is currently the subject of much debate. Similarly, the use of adjuvant chemical treatments has been validated by numerous articles, with chlorhexidine receiving the most substantial support (McGrath et al. 2023; Feldman et al. 2022). In contrast, advances in biotechnology have introduced bioadhesive gels that can prevent biofilm formation by ensuring prolonged contact with the oral cavity and the release of antimicrobial agents (Sterzenbach et al. 2020).

Furthermore, the topography, roughness, and chemical composition of the implant surface play an important role in the development and establishment of biofilm and in the efficacy of peri‐implantitis treatment. For this reason, these surfaces have been improved over the years to ensure that they can be sanitized in the event of colonization without compromising the implant's osseointegration (Yan Lin et al. 2013).

This research aims to evaluate the efficacy of a new bioadhesive gel in preventing microbial biofilm formation on different surfaces of dental implants through a microbiological analysis of the gel's effect on inhibiting the growth and adhesion of a simplified consortium of pathogenic microorganisms related to peri‐implantitis.

## 2 Materials and Methods

### 2.1 Titanium Disks and Surface Treatments

The study was carried out on titanium disks made by Klockner S.L. (Barcelona, Spain). All the disks were made of a titanium alloy OPTiMUM grade IV cp titanium, which undergoes a cold‐drawing treatment, the same alloy used to manufacture the brand's dental implants.

**Figure 1:**
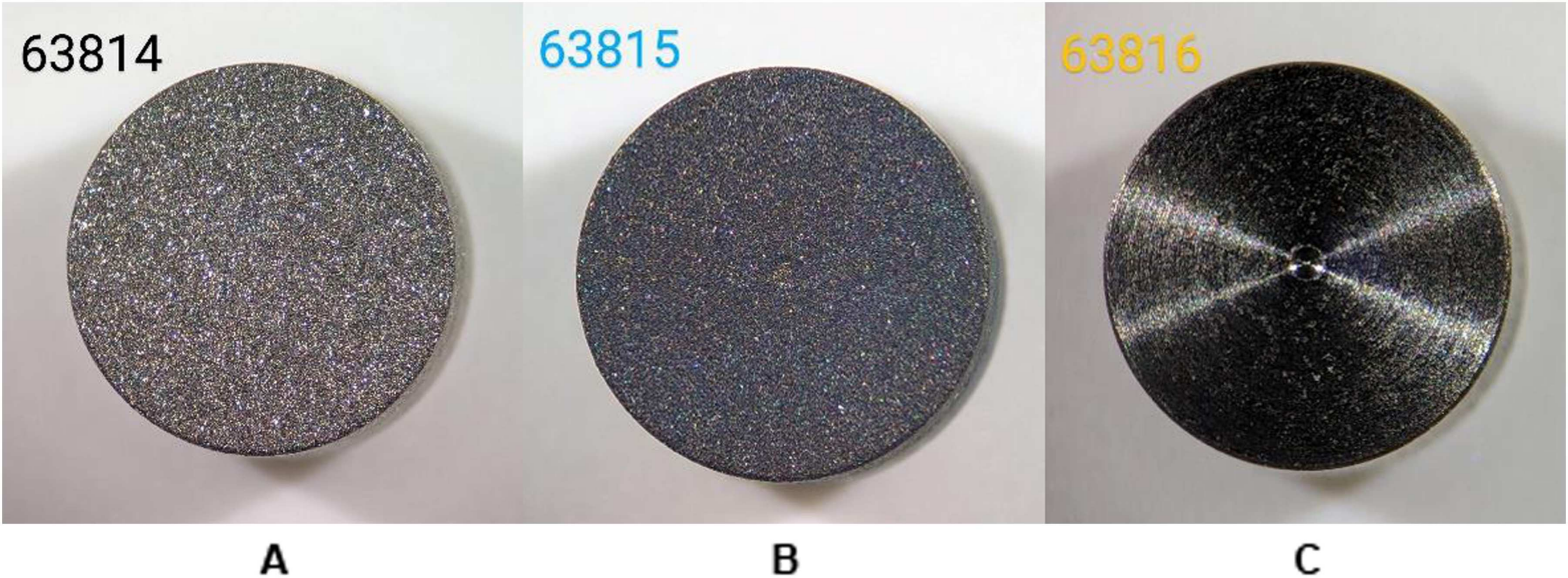
Photographs of the study disc surfaces: (A) Sandblasted surface. (B) ContacTi surface. (C) Machined surface.

The disks were classified according to their physicochemical properties resulting from the different surface treatments (Figure 1):

- Sandblasted surface: Particle bombardment blasts the surface with aluminum oxide. This treatment gives the implant a moderate roughness with a Sa of 1.6 ± 0.1 µm, increasing the titanium's surface hardness. Subsequently, acid passivation is carried out to protect the implant from ionic exchange with the medium and thus from material corrosion. This increases the contact surface between bone and implant, optimizing osteoblast adhesion and inhibiting bacterial adhesion.
- ContacTi surface: thermochemical treatment consisting of an alkaline immersion and a heat treatment. This creates a stable titanate layer on the surface. The negative charge of this layer accelerates the osseointegration process for two reasons:
  ◦ Patient's appetite: The dense, negatively charged titanate layer directs the migration of cations, calcium, and phosphate to the surface. Progressively, a spontaneous apatite layer forms, which is ionically bound to the implant. It is essential to note that the crystalline apatite layer that forms is the patient's own and comparable to the bone's natural hydroxyapatite.
  ◦ Specific protein adsorption: The negative potential conferred by the titanate layer promotes the adsorption of proteins that promote bone formation. ContacTi provides osteoconductive properties to the implant and accelerates the osseointegration process.

- Machined surface: a smooth and polished surface obtained by machining.

All disks used in the study were sterilized with ethylene oxide.

### 2.2 Bioadhesive Gel

The bioadhesive gel tested in the study was Lacer Chlorhexidine (LACER S.A.U, Barcelona, Spain). The gel's composition is detailed in Table 1.

**Table 1:** Lacer chlorhexidine bioadhesive gel composition.

**Ingredients**	**% (m/m)**
Toothpaste aroma 1/074569	0.12
Chlorhexidine digluconate 20%	1.06
O‐cymen‐5‐ol	0.10
Purified water	71.047
Sucralose	0.07
Propylene glycol	10.00
Vanilla	0.003
Menthol crystal	0.10
Benecel K4M (hydroxypropyl methyl cellulose)	3.20
Glycerin	7.00
Sorbitol (bulk)	7.00
Peg‐40 hydrocastor oil (cosmetics)	0.30

*Note:* pH = 5.9–7.2; density at 20°C: 1.052 g/mL ± 0.010 g/mL (1.042–1.062 g/mL).

The gel was applied manually to the surface of the disks, distributing 0.1 g of gel uniformly per implant.

### 2.3 Simplified Microbial Consortium

The microbial consortium used in the study represents a simplified microbial community associated with peri‐implantitis processes, considering that creating a model that accurately reflects clinical conditions is very complex. Other authors have previously defined this microbial model, which has been used in microbiological studies on dental implants (Bermejo et al. 2019a; 2019b), taking into consideration the clinical limitations this entails.

The consortium is composed of the reference strains *Streptococcus oralis* CECT 907T, *Veillonella parvula* NCTC 11810 (=DSM 2008), *Actinomyces naeslundii* ATCC 19039 (=DSMZ 17233), *Fusobacterium nucleatum* DMSZ 20482, *Aggregatibacter actinomycetemcomitans* DSMZ 8324, and *Porphyromonas gingivalis* ATCC 33277 (=DSMZ 20709).

All microorganisms were initially cultured on blood agar plates (Blood Agar Oxoid No 2) supplemented with 5% (v/v) sterile horse blood (Oxoid), 5 μg/L haemin (Sigma), and 1.0 mg/L menadione (Merck). Culture was performed under anaerobic conditions (10% H_2_, 10% CO_2_, and 90% N_2_ equilibrium) at 37°C for 24–72 h.

To inoculate the disks and conduct the biofilm formation study, pure cultures of each bacterium were grown under anaerobic conditions in modified brain‐heart infusion medium (BHI, Becton, Dickinson and Company). This medium was supplemented with 2.5 g/L mucin (Oxoid), 1 g/L yeast extract (Oxoid), 0.1 g/L cysteine (Sigma), 2 g/L sodium bicarbonate (Merck), 5 mg/L hemin (Sigma), 1 mg/L menadione (Merck), and 0.25% (v/v) glutamic acid (Sigma). After 24 h, each bacterial culture was quantified spectrophotometrically (OD600). Subsequently, the cultures were mixed at a concentration determined to emulate a natural microbial consortium (Table 2).

**Table 2:** Microbial composition of the model consortium used in the study.

**Strain**	**Cell concentration (CFU/mL)**
*Streptococcus oralis* CECT 907^T^	10^3^
*Actinomyces naeslundii* ATCC 19039	10^5^
*Veillonella parvula* NCTC 11810	10^5^
*Fusobacterium nucleatum* DMSZ 20482	10^6^
*Aggregatibacter actinomycetemcomitans* DSMZ 8324	10^6^
*Porphyromonas gingivalis* ATCC 33277	10^6^

### 2.4 Disk Inoculation

Sterile disks were placed in wells of 24‐well tissue culture plates (Ref. 83,3922,500, Sarstedt, SARSTEDT S.A.U, Barcelona, Spain) and then inoculated with 900 μL of the microbial consortium suspension.

Subsequently, the culture plates were incubated at 37°C under anaerobic conditions (5% H_2_, 5% CO_2_, and 90% N_2_ equilibrium) for 10 days. Wells without disks containing the culture medium were used as a control.

**Figure 2:**
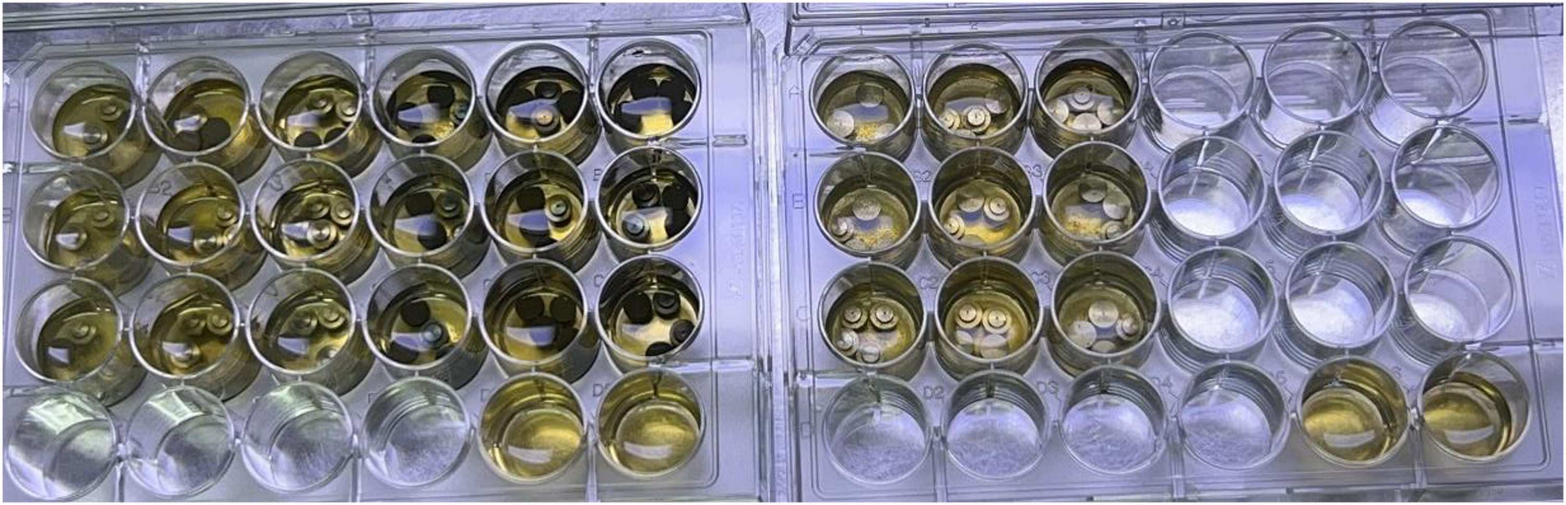
Multiwell plates were used to incubate the microbial consortium on implants treated and untreated with bioadhesive gel.

Three biological replicates were tested for each type of implant surface (Sandblasted, ContacTi, and Machined) and treatment (with and without bioadhesive gel) (Figure 2).

### 2.5 Count of Culturable Micro‐Organisms

To determine the viability of the microorganisms after treatment with the bioadhesive gel, the number of Colony‐Forming Units (CFU) per disc was counted. This metric reflects the number of viable cells capable of forming biofilm after a 10‐day incubation period.

After incubation, the disks were removed from the multiwell plates using sterile forceps and transferred to Eppendorf tubes containing PBS buffer. The Eppendorf tubes were subjected to intense vortexing to dislodge the biofilm adhering to the implants. The resulting bacterial suspensions were seeded on blood agar plates (Blood Agar Oxoid No 2) spiked with 5% (v/v) sterile equine blood (Oxoid), 5 μg/L haemin (Sigma), and 1.0 mg/L menadione (Merck). The culture was performed under anaerobic conditions (5% H2, 5% CO_2_, and N_2_ balance) at a constant temperature of 37°C for a period varying between 24h and 72 h.

### 2.6 Quantification of Developed Biofilm

The biofilm developed on the surface of the implants after incubation with the microbial consortium was quantified using the crystal violet technique (Shih 2021). At the end of the incubation time of the microbial consortium with the implants, the implants were collected with sterile forceps. The exopolysaccharides of the biofilms were stained with crystal violet in 96‐well microtiter plates.

The disks were washed twice with sterile PBS buffer (200 μL/well) to remove any remaining bacterial cells not attached to the biofilm.

The PBS was then removed, 200 μL of methanol was added to fix the biofilms to the disc surface, and the mixture was incubated for 60 s at room temperature.

Subsequently, the methanol was removed with a pipette, and the disks were left to dry in a laminar flow cabinet for 10–15 min. After drying, 200 μL of prepared 0.5% (w/v) crystal violet was added and incubated for 30 min at room temperature.

After this time, the volume of the crystal violet suspension was removed, and the disks were washed 3–5 times with PBS (200 μL) until no detached crystal violet was observed. To release the crystal violet adhering to the exopolysaccharide matrix of the biofilms, 200 μL of methanol was added again and incubated at room temperature with gentle agitation for 30 min. The amount of crystal violet attached to the biofilm was proportional to the amount of organic matter. The released crystal violet was quantified with a plate reader at a wavelength of A590 nm (Tecan Infinite 200 PRO).

### 2.7 Microscopic Characterization of the Biofilms by SEM

Scanning electron microscopy (SEM) was used to analyze the structural and morphological characteristics of the biofilms developed on the disks.

The implants were fixed by overnight immersion in Karnovsky's fixative (2% paraformaldehyde and 2.5% glutaraldehyde in 0.1 M sodium phosphate buffer at pH 7.4). They were then washed with sterile deionized water and a series of ethanol solutions (30%, 50%, 70%, 90%, and 100% absolute ethanol [PanReac, AppliChem; Panreac Química S.L.U., Barcelona, Spain]).

Samples were incubated in a desiccator for 48 h before being embedded in resin with carbon tape. Sputtering was performed with gold and palladium particles, and the samples were examined with a Hitachi S4800 field emission scanning electron microscope (FE‐SEM).

### 2.8 Metataxonomic Characterization of Biofilms

The massive amplicon sequencing technique was used to characterize the taxonomic profile of the bacterial community of the biofilms developed in each disc. Metagenomic DNA was extracted using the DNeasy PowerSoil Pro kit (QIAGEN GmbH, Ref. 47014) following the manufacturer's protocol and subsequently quantified with the Qubit 2.0 fluorometer (Qubit 1X dsDNA HS Assay kit, Thermo Fisher, USA). Once the presence of DNA in the samples was verified, libraries were prepared according to Illumina's standard protocol. The amplicons were sequenced using the Illumina HiSeq platform (2×300 bp). The amplification protocol and library preparation description can be found in Satari et al. (2020). The raw sequences generated by Illumina were imported into the bioinformatics tool Qiime2 (Bolyen 2019 to perform an initial quality control process of the sequences with DADA2. Taxonomic assignment of each amplicon sequence variant (ASV), defined at 99.9% sequence similarity, was performed using the classify‐Sklearn module in combination with the SILVA v138 database (Quast 2012). Microbial ecology analyses and statistical tests were performed using different R packages, including Phyloseq (McMurdie 2013) and Vegan (Oksanen 2015) Abundance data were normalized with the total sum normalization (TSS) approach to minimize potential bias arising from different sequencing depths between samples. Differential abundance analyses (DAA) of taxa were performed using the MaAsLin2 R package (v. 1.0.0) (Mallick 2021) with the following parameters: min_abundance = 0, min_prevalence = 0.5, max_significance = 0.05, normalization = ‘None’, transform = ‘LOG’, analysis_method = “LM”, correction = “BH”, and standardize = FALSE.

### 2.9 Statistical Analysis

Quantitative data obtained from colony‐forming unit (CFU) counts were analyzed to assess differences between implant surfaces and treatment conditions (with and without bioadhesive gel). Data were expressed as mean ± standard deviation of three biological replicates.

Statistical comparisons between groups were performed using one‐way analysis of variance (ANOVA) followed by Tukey's post hoc test for multiple comparisons. A *p*‐value < 0.05 was considered statistically significant.

Statistical analyses were performed using R software (version 4.3.2).

For metataxonomic analyses, statistical evaluation of differential abundance was conducted using the MaAsLin2 R package, applying linear models with Benjamini–Hochberg correction for multiple testing, as described above.

## 3 Results

### 3.1 Count of Micro‐Organisms

After 10 days of incubation, statistically significant differences were observed between implants treated with bioadhesive gel and untreated implants. Complete inhibition of microbial growth and biofilm formation was observed in gel‐treated implants (*p*‐value < 0.05).

Regarding the type of surface, the ContacTi surface showed higher bacterial viability than the machined and sandblasted surfaces, which did not show significant differences.

**Figure 3:**
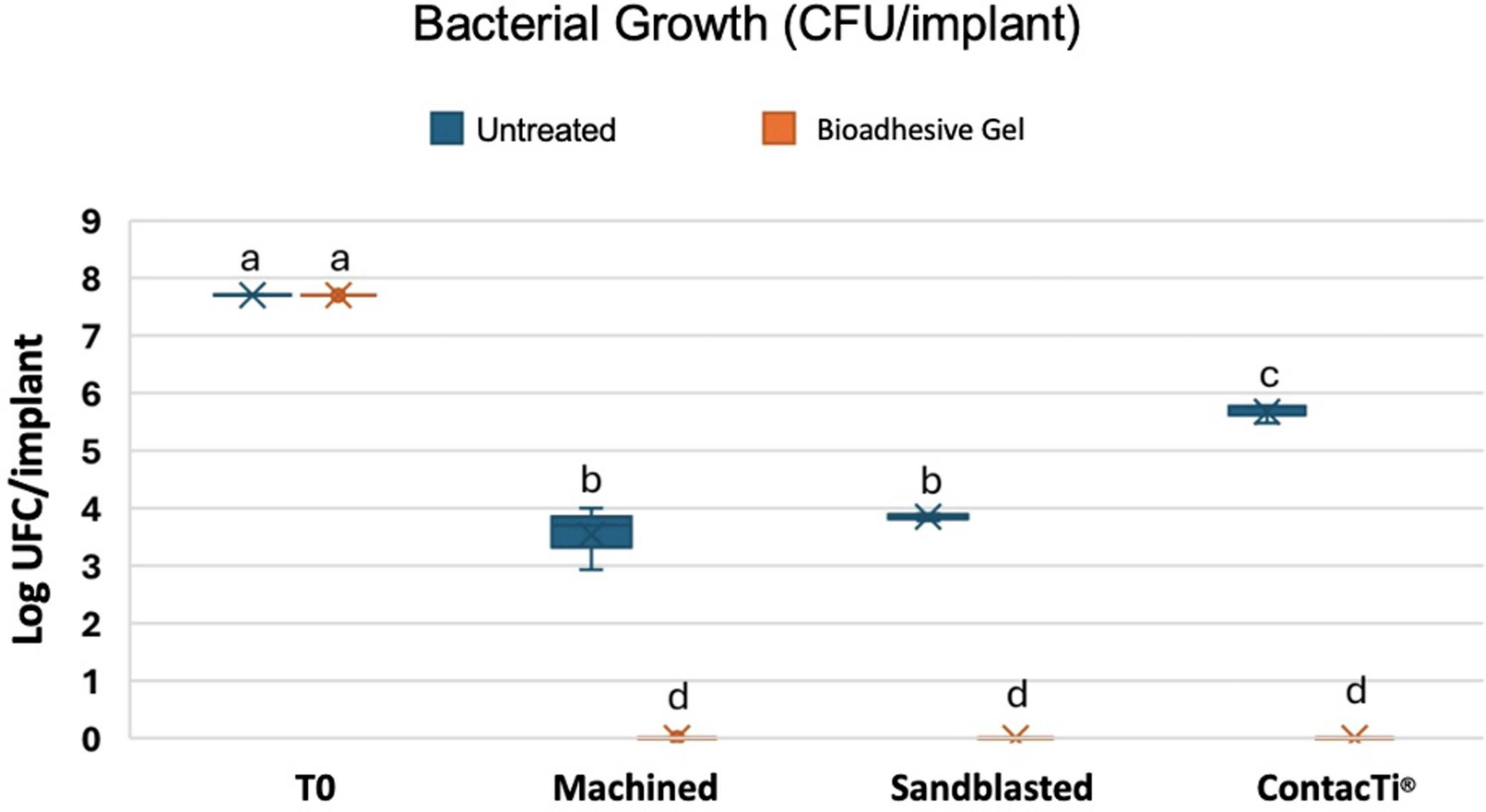
Colony Forming Unit (log) counts per implant tested. Different letters indicate significant differences between data according to Tukey's test (*p*‐value < 0.05).

In all cases, the microbial community value at T0 was higher than at T10 (T0 being the bacterial concentration of the supernatant after inoculation and T10 being the viable cells that formed biofilm instead of remaining in the supernatant) (Figure 3).

### 3.2 Quantification of Developed Biofilms

**Figure 4:**
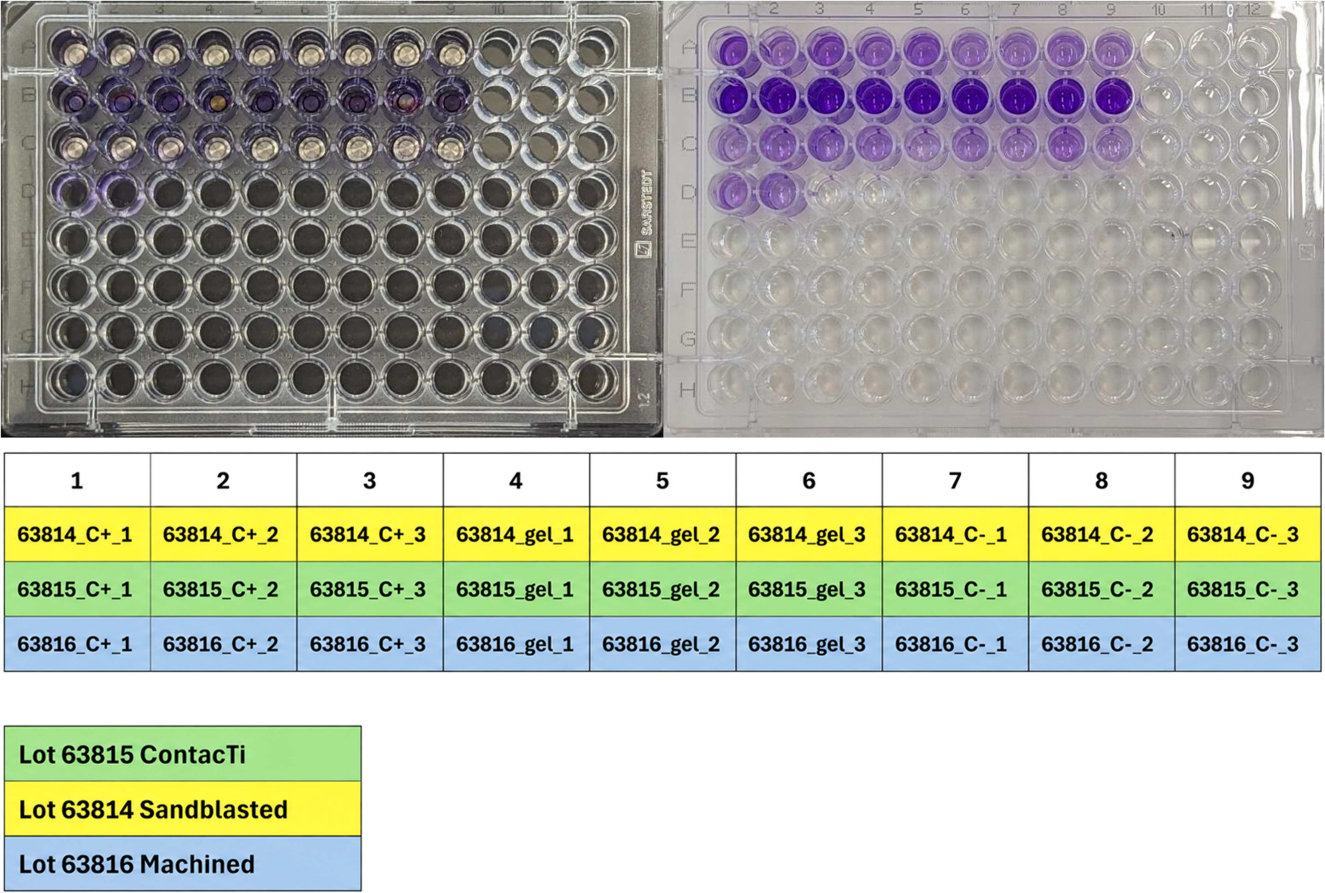
An assay to quantify the amount of biofilm formed on the surface of the implants by staining with crystal violet. The left plate shows the implants after staining with the dye. The right plate shows the density of crystal violet released from the implants, which was invalid for this study. The lower part shows the arrangement of the samples in the test.

After using the crystal violet staining technique for this type of sample, it was concluded that it was unsuitable for this model, as crystal violet is used to quantify bacterial biofilms on surfaces of inert materials; the dye binds to the glycopolysaccharide matrix through interactions of negative and positive charges. The problem is that the implants, specifically the ContacTi surface, have many negative charges, so the dye adheres to the surface regardless of the presence of biofilms. The implants’ negative charge and ContacTi are intentionally introduced to attract Ca^2^+ cations and promote osseointegration. This non‐specific binding of crystal violet to the implant surface was observed in the negative controls, and the test samples showed comparable values, thus invalidating the technique (Figure 4).

### 3.3 Microscopic Characterization of the Biofilms by SEM

By observing the surface of the implants, the amount of biofilm developed by the microbial consortium after 10 days of incubation could be determined semi‐quantitatively.

Those implants treated with bioadhesive gel did not show biofilm development due to the inhibitory capacity of its active ingredients. In some cases, planktonic (loose, non‐biofilm‐forming) bacteria were observed. As no biofilm‐associated structure was observed, it was hypothesized that inhibition occurred in the early stages after inoculation.

On the other hand, the untreated implants did show biofilm development, with differences in the degree of biofilm maturation between the three surfaces tested.

**Figure 5:**
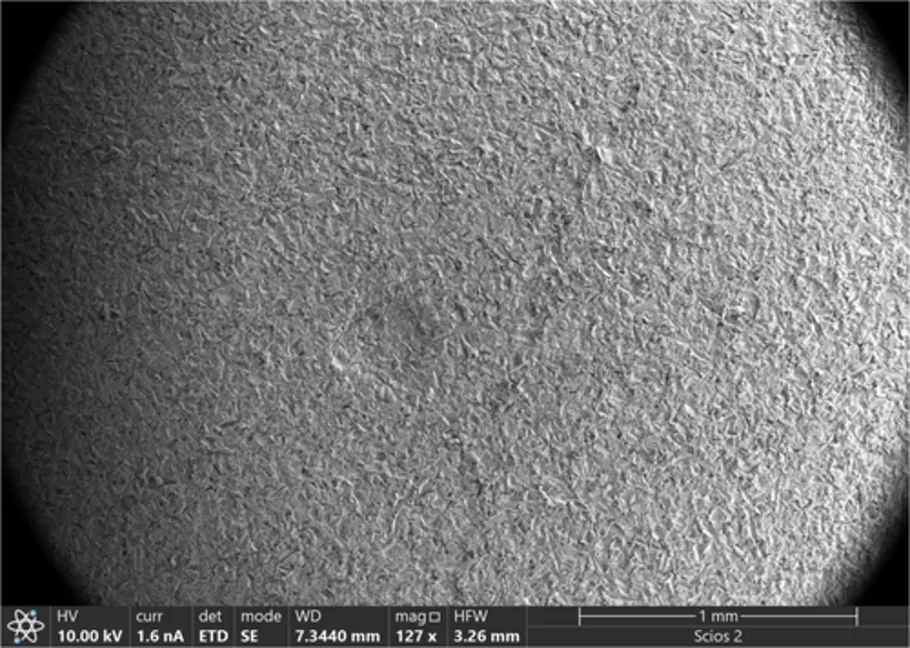
Microscopic characterization of the surfaces by SEM. Uninoculated and untreated sandblasted surface.

**Figure 6:**
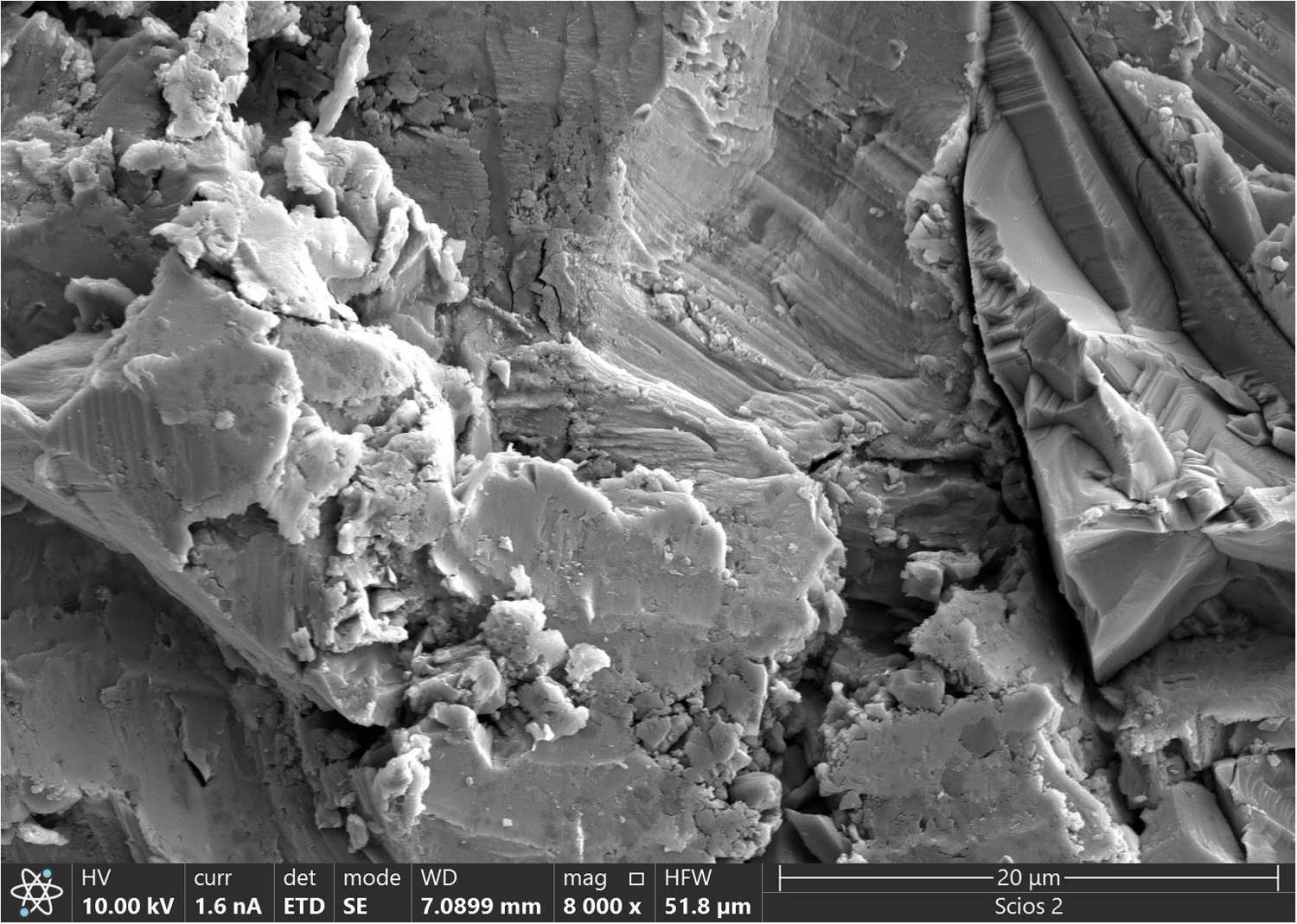
Microscopic characterization of the surfaces by SEM. Uninoculated and untreated sandblasted surface.

**Figure 7:**
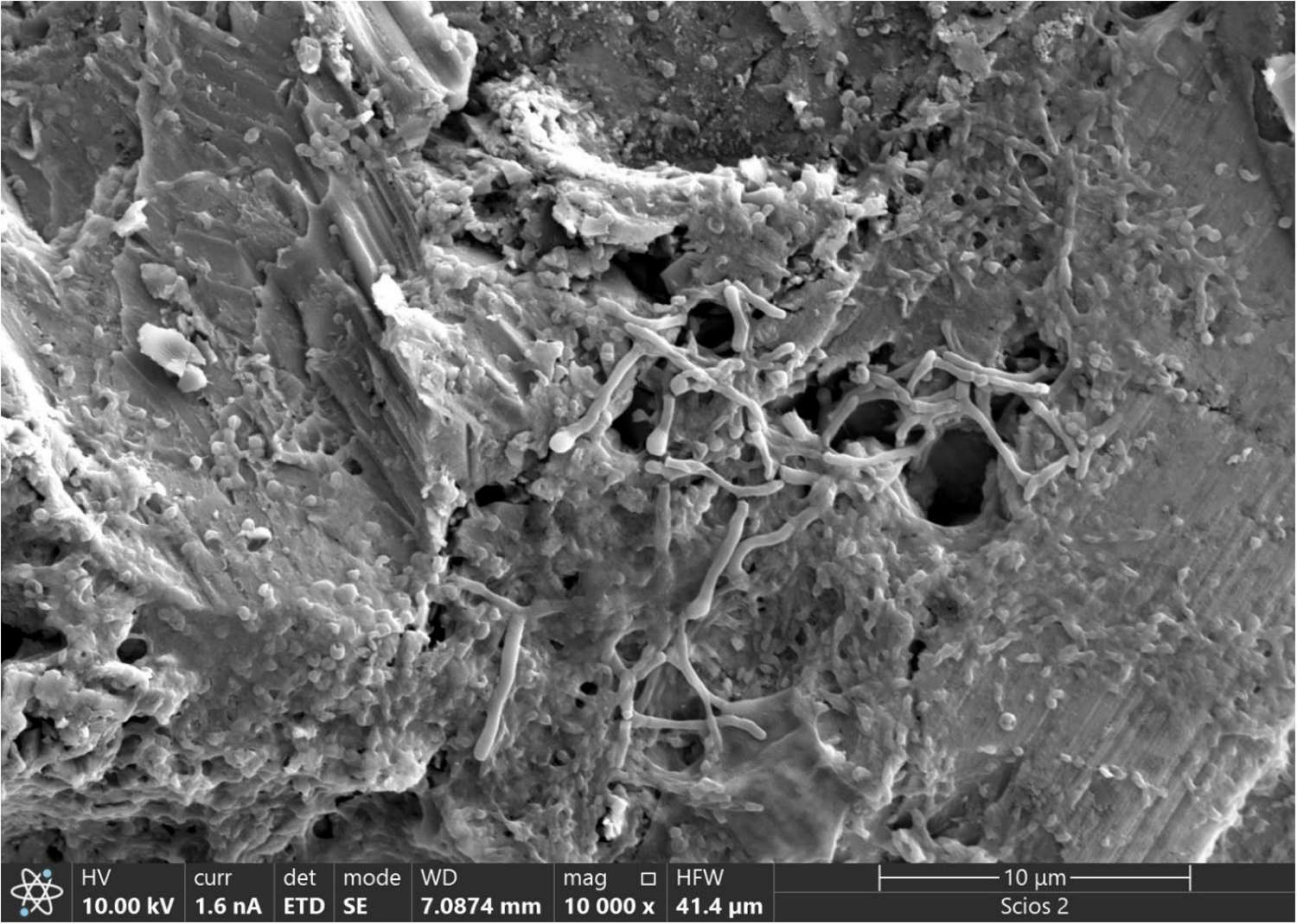
Microscopic characterization of the surfaces by SEM. Sandblasted surface inoculated and not treated with bioadhesive gel.

**Figure 8:**
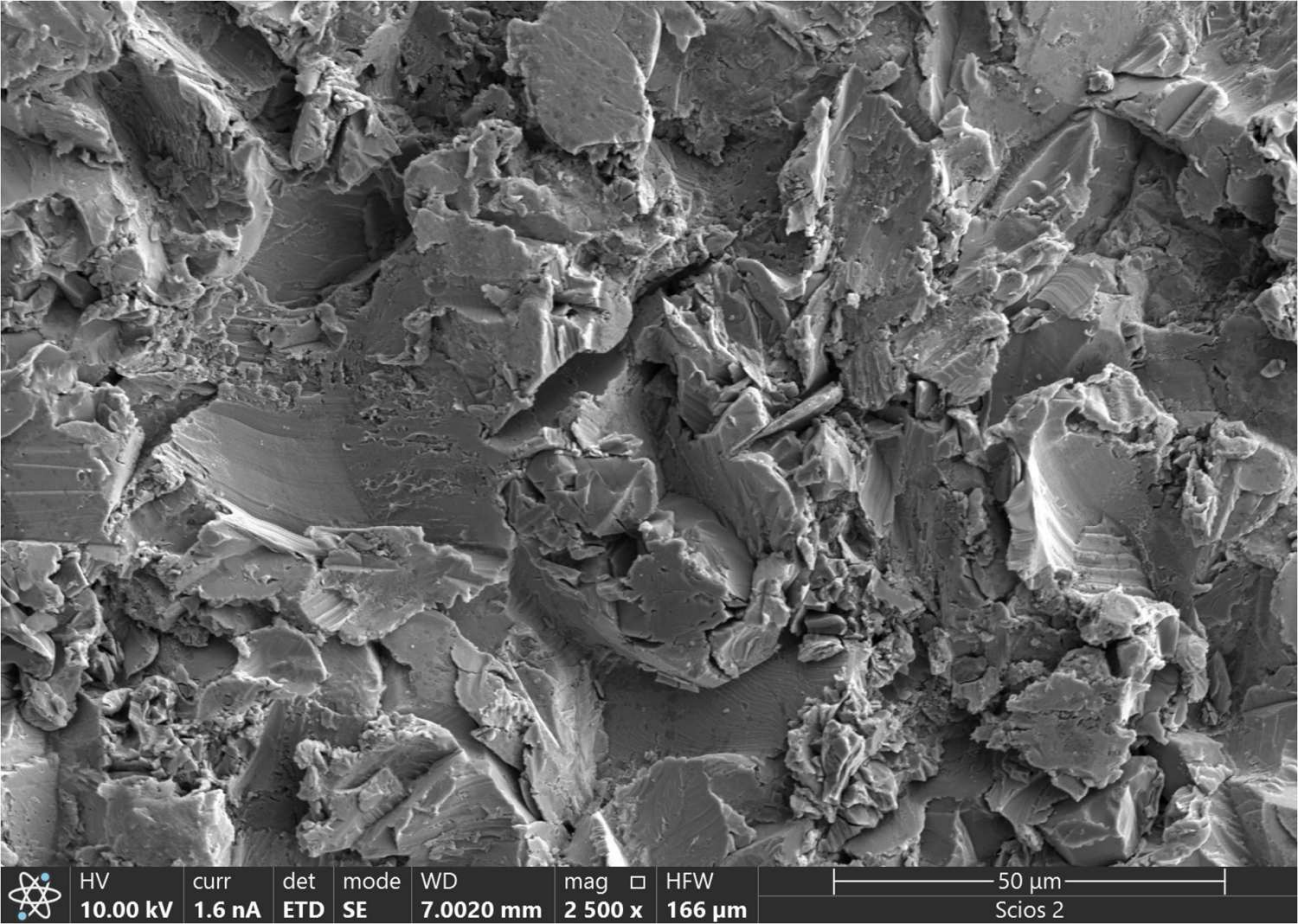
Microscopic characterization of the surfaces by SEM. Inoculated sandblasted surface treated with bioadhesive gel.

On untreated sandblasted implants: dense biofilm with different morphotypes in its composition, including cocci corresponding to *S. oralis* and/or *V. parvula*, and bacilli that could be identified as *A. naeslundii*, *F. nucleatum*, *A. actinomycetemcomitans*, and/or *P. gingivalis* (Figures 5, 6, 7, 8).

**Figure 9:**
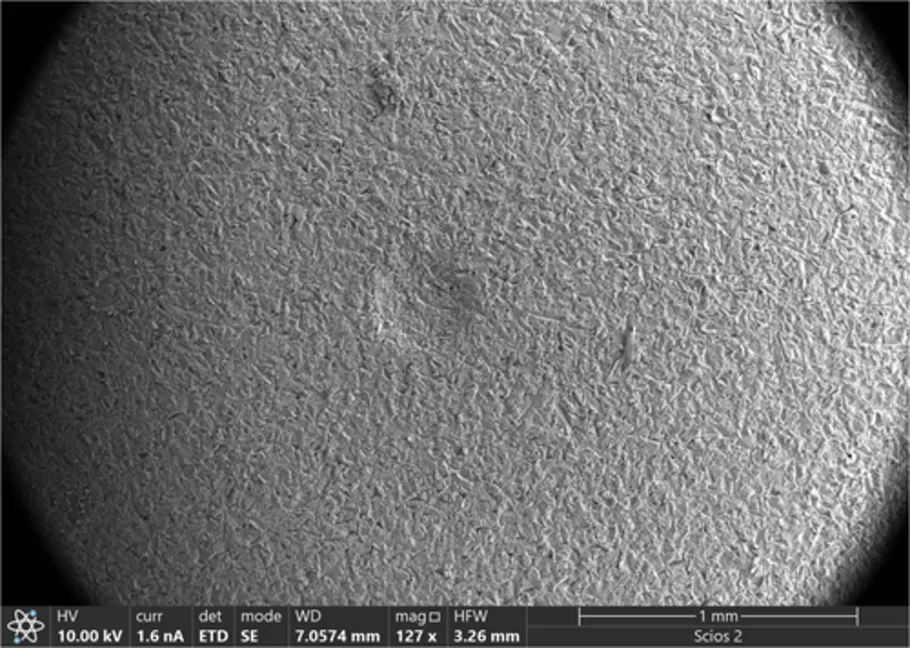
Microscopic characterization of the surfaces by SEM. Uninoculated and untreated ContacTi surface.

**Figure 10:**
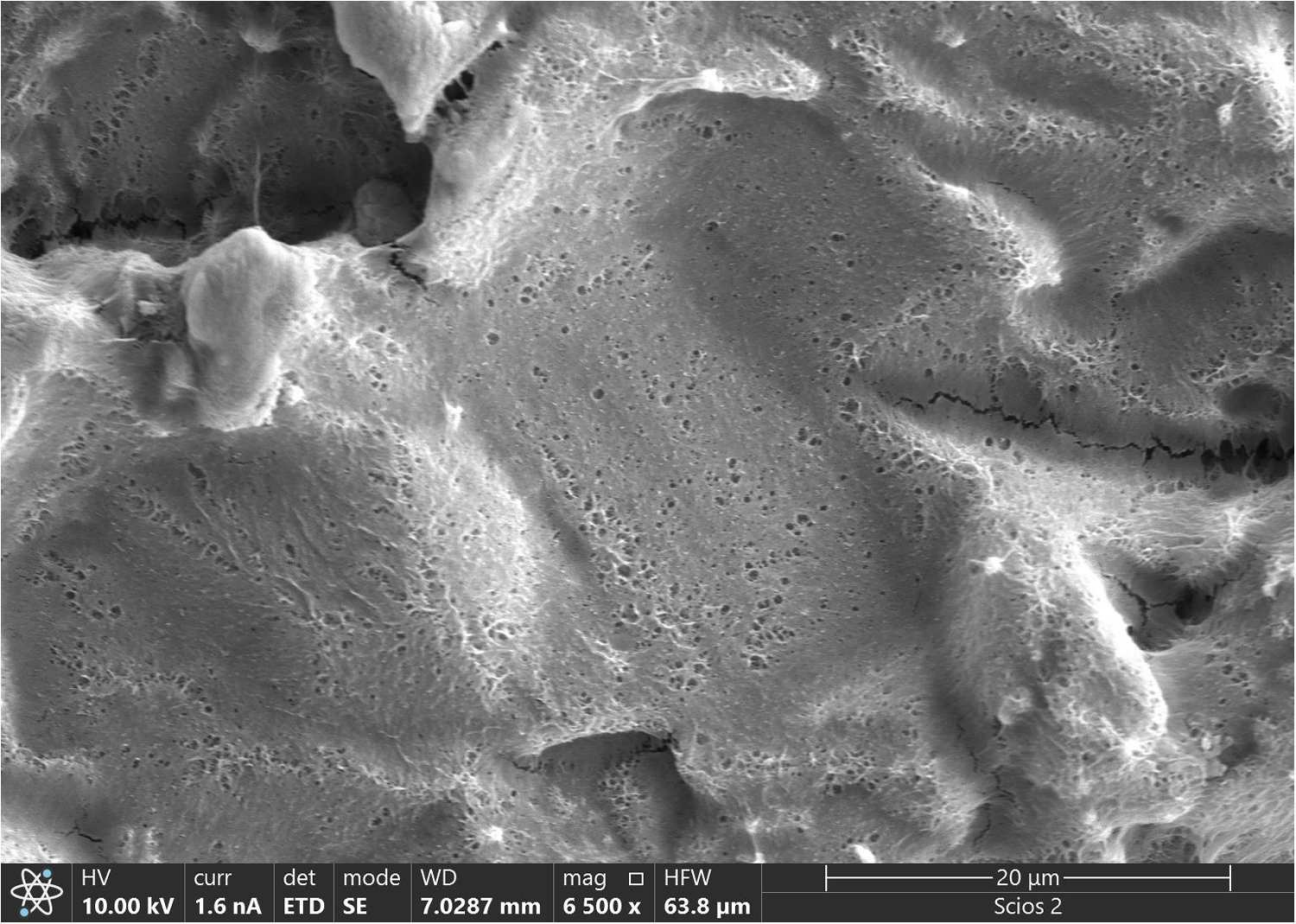
Microscopic characterization of the surfaces by SEM. Uninoculated and untreated ContacTi surface.

**Figure 11:**
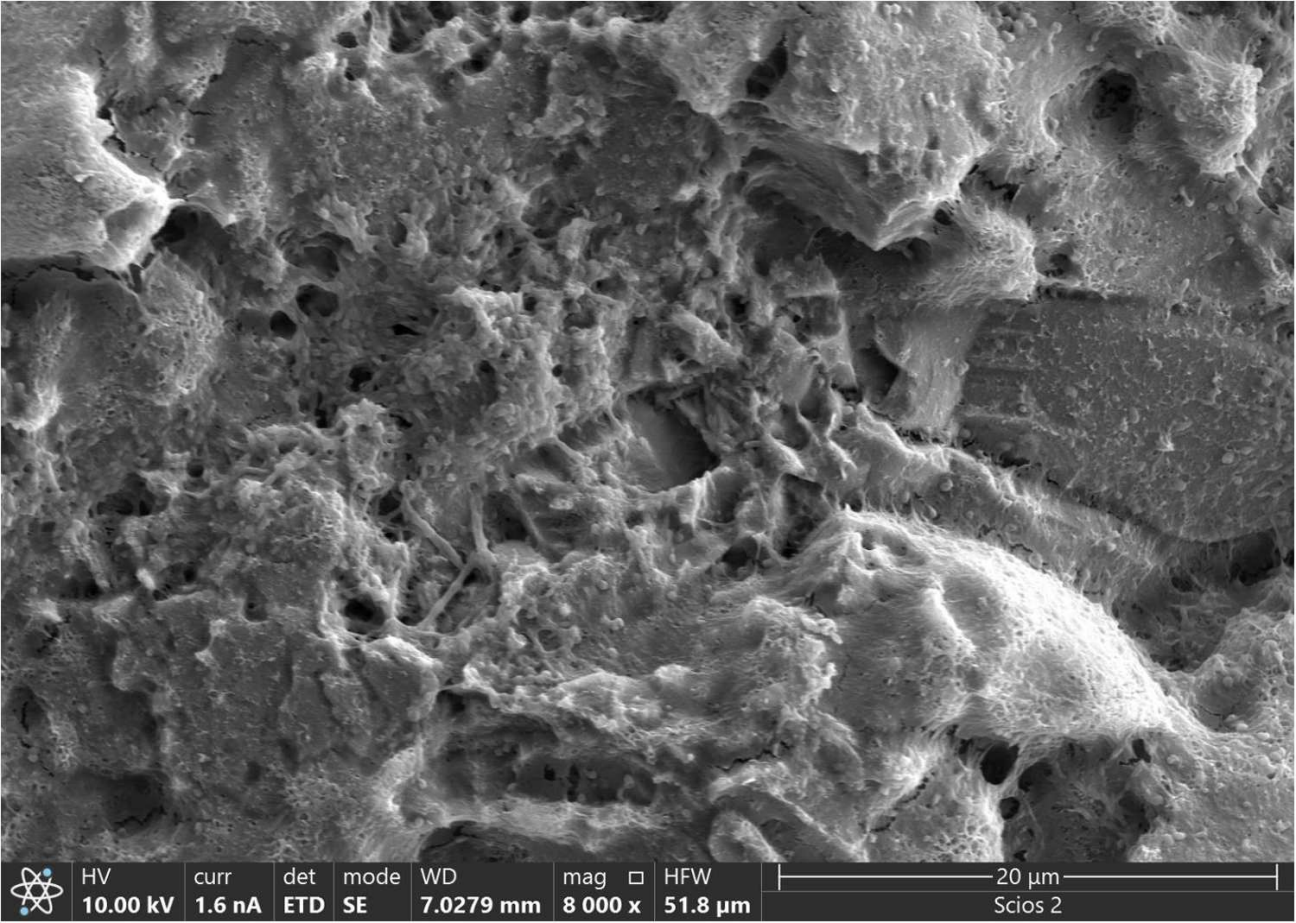
Microscopic characterization of the surfaces by SEM. Inoculated and untreated ContacTi surface.

**Figure 12:**
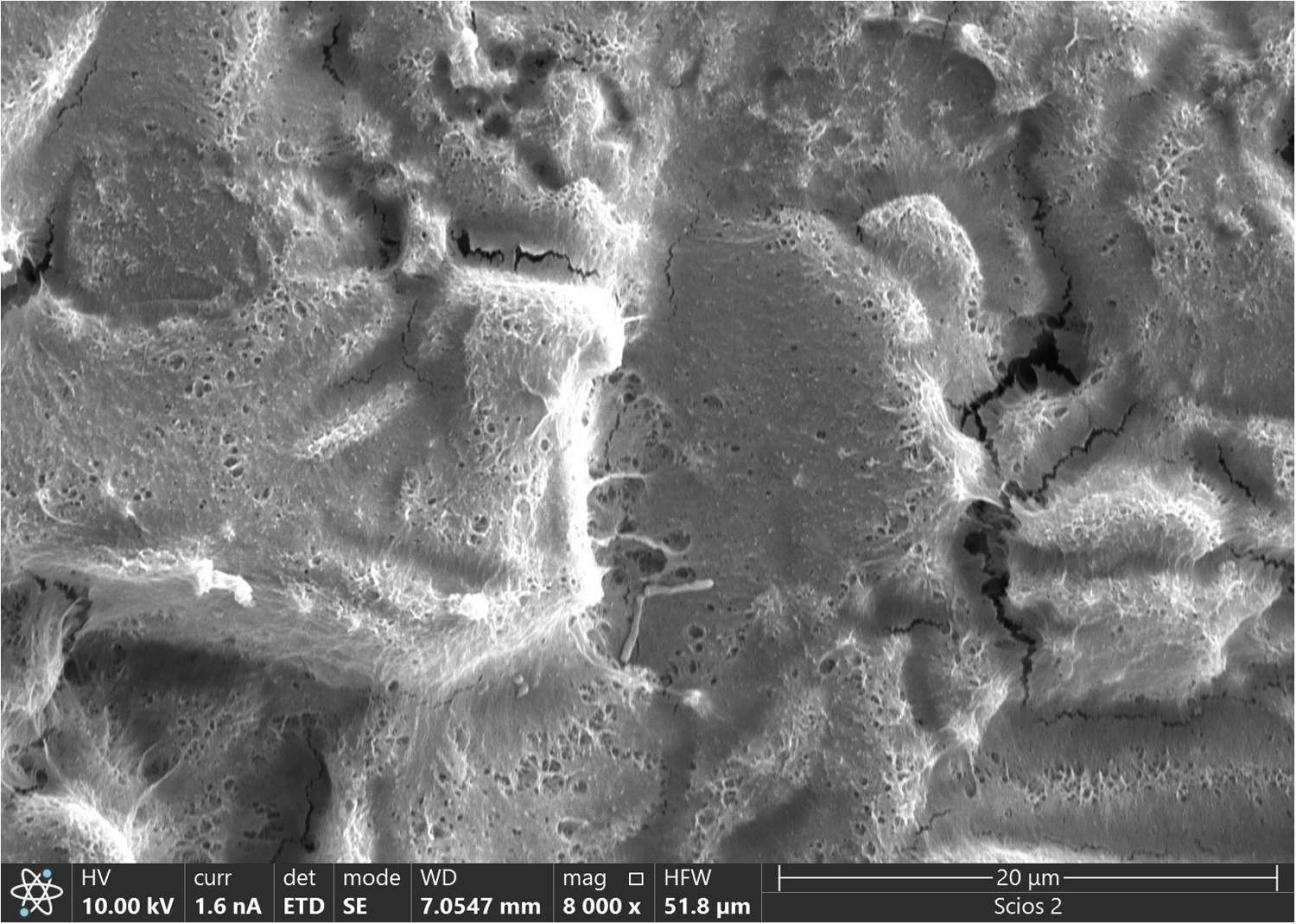
Microscopic characterization of the surfaces by SEM. Inoculated and treated the ContacTi surface.

On untreated ContacTi implants, there is slightly less dense biofilm than on sandblasted and machined implants; however, the viability of bacteria associated with this surface is slightly higher (Figures 9, 10, 11, 12).

**Figure 13:**
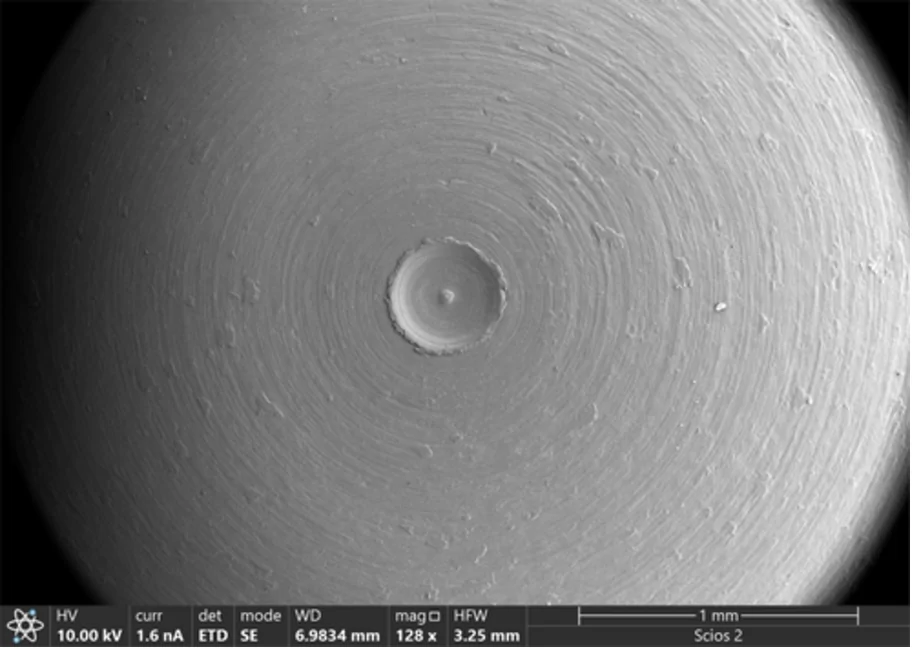
Microscopic characterization of the surfaces by SEM. Uninoculated and untreated machined surface.

**Figure 14:**
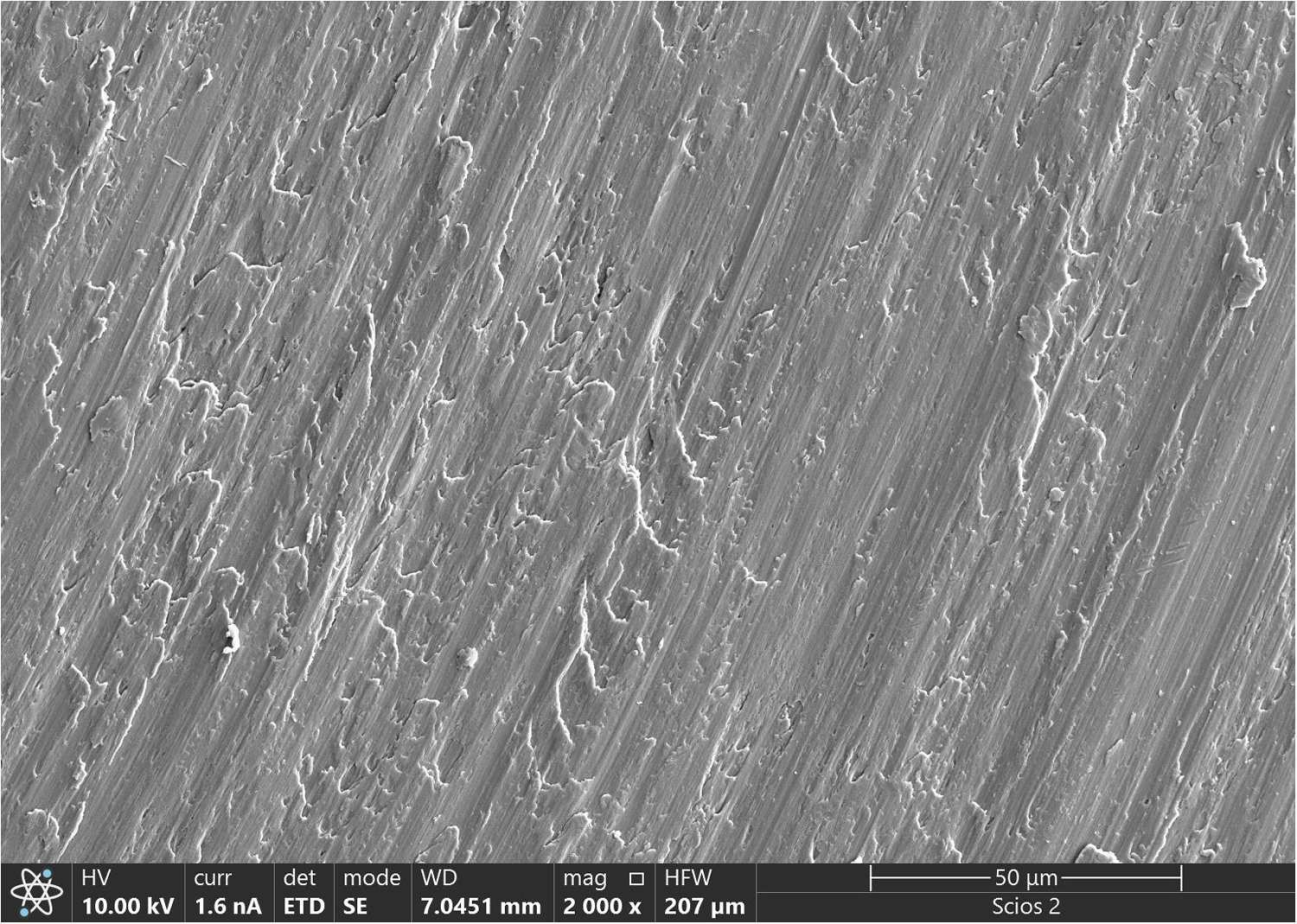
Microscopic characterization of the surfaces by SEM. Uninoculated and untreated machined surface.

**Figure 15:**
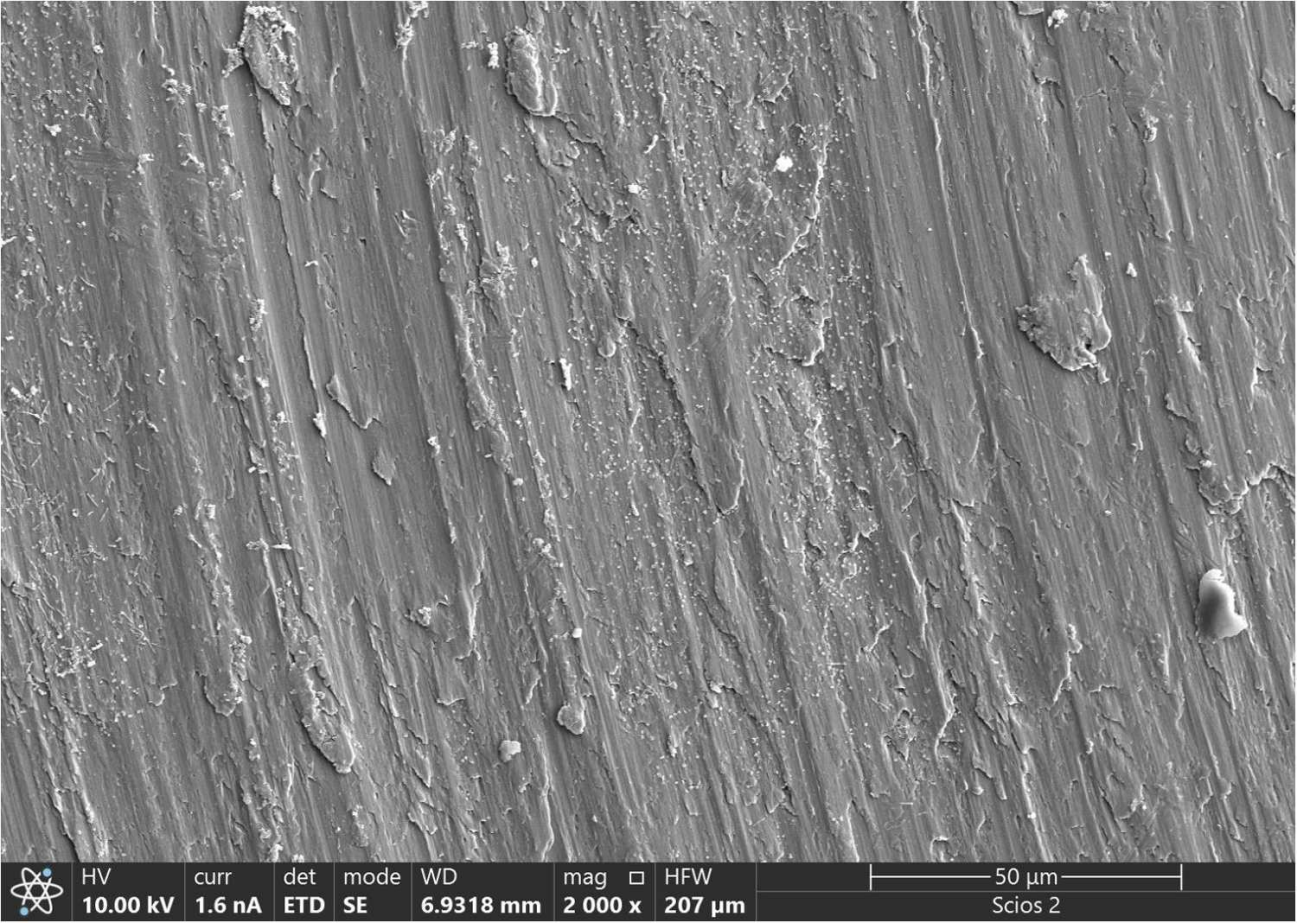
Microscopic characterization of the surfaces by SEM. Inoculated and untreated machined surface.

**Figure 16:**
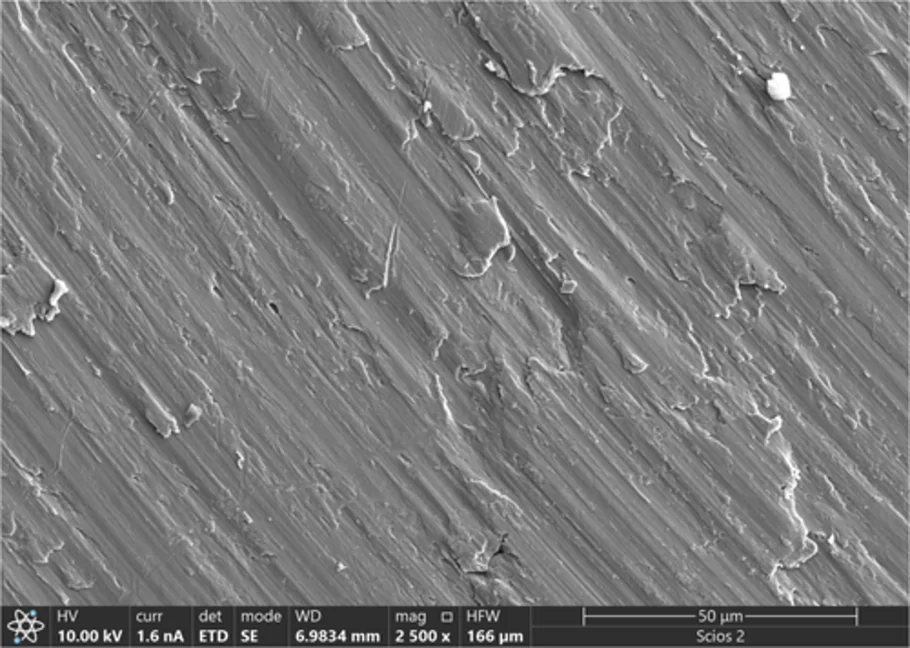
Microscopic characterization of the surfaces by SEM. Inoculated and treated machined surface.

In untreated machined implants, the biofilm formed occupies a large surface area, although it is characteristically three‐dimensionally sparse, and coccoid species dominate (Figures 13, 14, 15, 16).

### 3.4 Impact of Surface Type and Bioadhesive Gel Use on Oral Microbiome Ecology

The microbial consortium consisted of *S. oralis, V. parvula, A. naeslundii, F. nucleatum, A. actinomycetemcomitans*, and *P. gingivalis*. Differences in microbial community composition between implant surfaces and treatments were studied using mass sequencing of 16S rRNA metagenomic gene amplifications.

The most abundant genera were *Aggregatibacter, Veillonella*, and *Fusobacterium*, while *Actinomyces*, followed by *Streptococcus*, were minority taxa in the community.

After 10 days of incubation, the original microbial community proportions were altered independently of surface type and treatment. The bioadhesive gel treatment had a more significant influence on changing the microbial community than the implant surface. Furthermore, in the implants not treated with bioadhesive gel, a greater cohesion between biological replicates was observed, in contrast to the treated implants, where there was greater dispersion. This suggests that the gel, despite impregnating the implants in the same way, had a variable effect on the microorganisms.

Notably, the diversity of the bacterial communities tended to decrease in all types of implants, regardless of whether or not they were treated with bioadhesive gel, which is associated with a marked decrease in some community members. This decrease was very marked in *P. gingivalis*, which went from values of 20% to 1%, suggesting that it developed difficulties in colonizing the biofilm formed, which was corroborated when the metagenomic analysis was carried out twice.

The surface of the implants influenced the microbial composition. The ContacTi surface significantly influenced biofilm development, with *Fusobacterium* and *Veillonella* genera increasing their relative abundances. At the same time, *Porphyromonas* and *Streptococcus* decreased their relative abundances (*p*‐value < 0.05), regardless of whether they were treated with gel or not. Non‐gel treatment showed an increase in *Actinomyces* and a decrease in *Aggregatibacter*, in contrast to gel treatment.

The implant with the sandblasted surface showed an increase in *Veillonella*. At the same time, *Porphyromonas* and *Streptococcus* decreased their relative abundances (*p*‐value < 0.05) regardless of whether they were treated with gel or not. However, the gel had a differential effect on this surface, particularly *Fusobacterium* and *Aggregatibacter*, which increased their relative abundances when treated with gel (*p*‐value < 0.05).

Finally, regardless of treatment, *Fusobacterium* and *Veillonella* increased their relative abundances on the machined surface, while *Porphyromonas* and *Streptococcus* decreased their relative abundances (*p*‐value < 0.05). When not treated with gel, an increase of *Actinomyces* was observed, and with the biocidal product, *Aggregatibacter* decreased.

**Figure 17:**
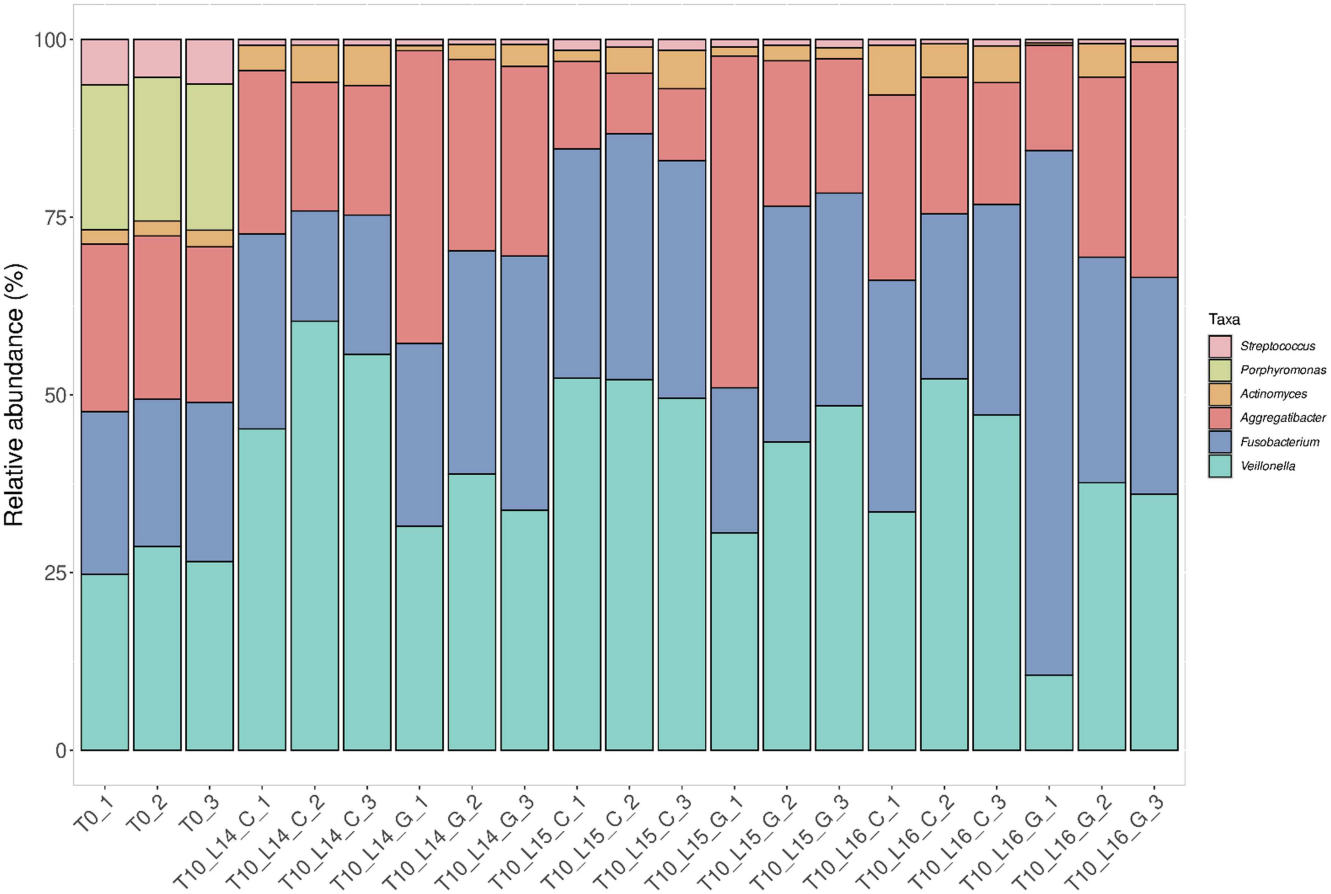
Taxonomic characterization of the microbial biofilm developed on the surface of untreated and bioadhesive gel‐treated implants. The relative values (%) obtained by the massive sequencing technique are shown. T0 corresponds to the initial inoculum. Two biological replicates were obtained from each sample. L14, sandblasted surface; L15, ContacTi surface; L16, machined surface.

The relative abundances of bacteria were much higher when the implants were treated with bioadhesive gel (Figure 17).

## 4 Discussion

Peri‐implantitis is a widely discussed pathology in implant dentistry, characterized by well‐defined clinical and radiographic signs. Its diagnosis is established by profuse bleeding on probing, a probing depth greater than 6 mm in one or more sites, and marginal bone loss greater than 3 mm compared with the radiograph taken at the time of implant loading. This last criterion is decisive in differentiating it from peri‐implant mucositis, in which there is no bone loss, as pointed out by Heitz‐Mayfield in his 2024 publication (Heitz‐Mayfield 2024).

Regarding the therapeutic approach, the literature classifies treatment strategies into two main groups: non‐surgical and surgical. Surgical therapies are subdivided into resective, regenerative, or combined techniques (Herrera et al. 2023). However, adjuvant treatments, such as local antiseptics, have been debated due to their questionable efficacy. According to a systematic review and meta‐analysis by Ramanauskaite et al. (2021), adding these antiseptics to mechanical therapy does not provide statistically significant clinical improvements, reaffirming that mechanical decontamination remains the mainstay of treatment for this pathology (Bermejo et al. 2019a, 2019b).

From a microbiological point of view, the present study employs a carefully selected microbial consortium, previously used in dental implant research and designed to accurately reflect actual clinical conditions. Unlike previous in vitro studies (Schmidt et al. 2017; Pereira et al. 2015), which relied on simplified models with only two bacterial species and incubation periods of up to 24 h, this study extends the incubation follow‐up to 10 days, providing a clinically relevant experimental model. Although the present study was conducted using a static in vitro biofilm model, which cannot fully reproduce the dynamic biological, immunological, and mechanical conditions present in the oral cavity, this approach allows strict experimental standardization and reproducibility. The polymicrobial consortium employed included early and late colonizers commonly associated with peri‐implantitis, providing a clinically relevant simplified model. Compared with previous in vitro studies based on mono‐species or dual‐species biofilms and short incubation periods (Schmidt et al. 2017; Pereira et al. 2015), the six‐species consortium and extended incubation time used in the present study offer a closer approximation to biofilm maturation processes. Nevertheless, extrapolation to clinical conditions should be performed with caution.

The results obtained show a differential behavior of bacteria depending on the implant surface (sandblasted, machined, or ContacTi) and the use or non‐use of bioadhesive gel treatment. Thanks to the microscopic characterization of the surfaces by scanning electron microscopy (SEM), all of these were analyzed.

In implants treated with this gel, a total inhibition of microbial growth and biofilm formation was observed, with statistically significant differences (*p* < 0.05) compared to untreated implants. Among the latter, the ContacTi surface showed a lower biofilm density, but with a higher bacterial viability compared to sandblasted and machined surfaces.

In the untreated sandblasted implants, the biofilm formed was dense. It showed a remarkable morphological diversity, including cocci compatible with *S. oralis* and/or *V. parvula*, as well as possibly identifiable bacilli such as *A. naeslundii, F. nucleatum, A. actinomycetemcomitans*, and *P. gingivalis*. The machined implants showed a more extensive biofilm on the surface, but a lower three‐dimensional density dominated by coccoid species. The biofilm was less dense in the untreated ContacTi implants than in the sandblasted implants, although bacteria were more viable (Figures 5, 6, 7, 8, 9, 10, 11, 12, 13, 14, 15, 16).

Focusing on the surface of the implants as the central focus of bacterial behavior, it should be noted that the biocompatibility of titanium is attributed to the formation of a stable and limited oxide layer on the surface that prevents the titanium from oxidizing and corroding (Saini 2015); this corrosion resistance allows the mechanical integrity of the material and the health of the peri‐implant tissue to be maintained. In addition, grade IV titanium, used in this study and most dental implants, has a lower modulus of elasticity (110 GPa) than other metals and has a higher fatigue strength, making it the material of choice (Niinomi 2003).

It is this same layer of titanium dioxide (TiO_2_) that confers the biocompatible property, which at the electrochemical and electrophysical level creates a microenvironment that causes the colonization of specific bacterial strains (*Streptococci* and *Actinomyces*), altering peri‐implant health (Lima et al. 2008). This has led to the need for physical, chemical, and biological surface modifications with the primary objective of minimizing biofilm formation without compromising osseointegration. Such changes range from macroscopic alterations to surface nanostructure adjustments, allowing for better interaction between the implant and the biological environment (Kligman et al. 2021). Finding the balance between achieving adequate osseointegration and decreasing biofilm formation is a challenge in implant surface design as a surface roughness of at least 1–1.5 μm is required to achieve osseointegration and as little as 0.2 μm to cause bacterial colonization (Teughels et al. 2006), the positive correlation between surface roughness and bacterial adherence has been demonstrated in in vivo and in vitro studies, where different implant surfaces are analyzed such as the study by Schmidlin et al. (2013); the latter surface was produced by blasting the surface with large grit particles (250–500 m) to remodel the macrostructure of the implant and then etching it with an acid to create micro‐irregularities (Barfeie et al. 2015); where it was determined that in the first 20 min bacterial colonization presented a similar average between all surfaces between 4.5 and 4.8 log CFU, until 16 h where the SLA surface presented the highest colonization values of 6.9 ± 0.2 CFU with a value of *p* < 0.05. With this in mind, methods to combat this bacterial adhesion have come onto the market as an adjuvant to mechanical debridement procedures, most of which are accompanied by treatment of the implant surface by polishing it (implantoplasty) (Dasgupta et al. 2023).

The use of local antibiotics has been widely discussed in the literature, as well as the use of Er: YAG laser and multiple bioadhesive gels, all accompanying surgical or non‐surgical mechanical debridement therapy. The systematic review and meta‐analysis by Baima et al. (2022) includes 16 RCTs with different clinical protocols for chemical and physical bacterial decontamination and found, with *p* < 0.05, that implantoplasty and the use of titanium brushes showed favorable results as the sole treatment method. In contrast, when combined with other adjuvant therapies the results were not statistically significant, in the case of the Er: YAG laser, the reduction in probing depth (PPD) was [−1.10; 0.63] with a *p*‐value = 0.59 and in the case of the use of local antibiotics (in this case amoxicillin and azithromycin) showed positive effects in terms of disappearance of bleeding and suppuration on probing (*p* < 0.008) but not in terms of reduction of PPD (*p* = 0.26). Therefore, neither showed superiority over the other regarding the definition of peri‐implant disease.

In the field of antibiotherapy in implant dentistry, even antimicrobial surfaces have been developed, type I surfaces that actively release drugs to prevent and eliminate bacteria whose effectiveness decreases over time and can lead to antibiotic resistance and type II surfaces, which have permanently bound antimicrobial agents, such as tetracycline and vancomycin (Hickok et al. 2018) For example, permanently coating implants with tetracycline, a bacteriostatic agent, effectively eliminates microorganisms that could contaminate the implant surface, aiding cell proliferation and thus more effective bone healing. The application of tetracycline on different implant surfaces, such as machined, sandblasted, or anodized, did not alter the surface microstructures that promote integration (Herr et al. 2008).

Finally, among adjuvant therapies, the use of bacteriostatic and bactericidal gels has been extensively analyzed, mainly in the treatment of periodontal patients (Lee and Nam 2022). Still, there is heterogeneity not only in the percentages of the components of the different gels but also in the components as such and in the follow‐up times, making it difficult to draw clear conclusions. The present study can clearly show differences between the different implant surfaces treated or not with bioadhesive gel. The treatment with bioadhesive gel had a more significant influence on altering the community of microorganisms than the implant surface, since in the implants not treated with bioadhesive gel a greater cohesion between biological replicates was observed, unlike those treated where there was a more excellent dispersion.

The surface of the implants did influence microbial composition. The ContacTi surface showed the most significant influence on the development of a biofilm; the genera *Fusobacterium* and *Veillonella* increased their relative abundances. At the same time, *Porphyromonas* and *Streptococcus* decreased their relative abundances (*p*‐value < 0.05), regardless of whether they were gel‐treated or not. Non‐gel‐treated implants showed an increase in *Actinomyces* and a decrease in *Aggregatibacter*. The implant with the sandblasted surface showed a rise in *Veillonella*. At the same time, *Porphyromonas* and *Streptococcus* decreased their relative abundances (*p*‐value < 0.05) regardless of whether they were treated with gel or not. However, the gel had a differential effect on this surface, particularly *Fusobacterium* and *Aggregatibacter*, which increased their relative abundances when treated with gel (*p*‐value < 0.05). Finally, regardless of treatment, Fusobacterium and Veillonella increased their relative abundances on the machined surface, while *Porphyromonas* and *Streptococcus* decreased their relative abundances (*p*‐value < 0.05). When not treated with gel, an increase of Actinomyces was observed, and with the biocidal product, *Aggregatibacter* decreased. No studies clearly demonstrate the use of bioadhesive gels on implant surfaces with such a sizeable microbial consortium.

Studies with different compounds such as the one by Iorio‐Siciliano et al. (2020) where they analyzed sodium hypochlorite gel “in vivo” in terms of probing depth found no statistically significant differences at 6 months follow‐up concerning the placebo group ([0.88 ± 1.04 mm] and [0.61 ± 0.75 mm with a *p*‐value < 0.58]), but they did obtain statistically significant results in terms of bleeding on probing (*p* = 0.0001).

Dini et al. (2025) published a highly relevant systematic review and meta‐analysis in which they analyzed the use of different bioadhesive gels to understand their various applications, carrying out a preclinical and clinical analysis of these gels, focusing on quantitative microbiological results, not only in dental implants but also in other orthopedic prostheses, all to pool results and alleviate the heterogeneity of published data and studies. The strains included are *S. aureus, S. mutans, A. actinomycetemcomitans, and P. gingivalis*, among others, as in the microbial consortium of our research, and the CFU data are analyzed after treatment of the surfaces with bioadhesive gels. Most of the studies used PEGDA (poly(ethylene‐glycol diacrylate); PEG (polyethylene glycol); chitosan and hyaluronic acid. Other gels contained vancomycin, minocycline, tetracycline, ampicillin, and others. This variety in components was derived from various microbiological entities and conditioned the selection of components. The gels oriented to the treatment of dental implants presented varied components oriented to eliminating anaerobic bacteria, presenting success rates between 95% and 100% with variable follow‐up periods. At this point, it is remarkable that chlorhexidine is considered the gold standard in terms of treating periodontal and periimplant disease; it might be an interesting yield of research of the present study how the analyzed microbial consortium behaves if the applied gel is not compound by chlorhexidine, so that we delimit the real role of each gel component. A limitation of the present study is the absence of a direct quantitative measurement of biofilm surface coverage on the implant disks. Although biofilm formation was evaluated through viable cell counts, metataxonomic profiling, and qualitative and semi‐quantitative structural assessment by scanning electron microscopy (SEM), precise quantification of the percentage of surface colonized by biofilm was not performed. An attempt to quantify total biofilm biomass using the crystal violet assay was carried out; however, this method proved unsuitable for the titanium implant surfaces evaluated, particularly for the ContacTi surface, due to non‐specific binding of the dye to negatively charged surfaces, resulting in background signals comparable to negative controls. Consequently, these data were excluded from quantitative interpretation. While SEM imaging provided valuable insights into biofilm presence, morphology, and maturation across different surface types and treatments, future studies should incorporate image‐based quantitative techniques, such as digital SEM image analysis or confocal laser scanning microscopy, to enable precise measurement of biofilm surface coverage and further strengthen the assessment of biofilm–implant interactions.

Another limitation relates to the experimental timing of bioadhesive gel application. The gel was intentionally applied prior to microbial inoculation to evaluate its preventive capacity against early bacterial adhesion and biofilm establishment rather than its therapeutic effectiveness on established infections. While this design allowed standardized evaluation of antimicrobial activity under controlled conditions, it does not fully replicate clinical scenarios in which complete implant surface coverage may be difficult to achieve, and prolonged gel contact cannot be guaranteed. Therefore, the results should be interpreted within the context of a preventive in vitro model.

This allows us to conclude that knowledge of the microbial spectrum is essential when creating a compound and that the “in vitro” phase of any study is necessary to establish percentages and predict microbial behavior. Although the present findings demonstrate promising antimicrobial and biofilm‐preventive effects under controlled in vitro conditions, future in vivo investigations and well‐designed clinical studies are required to confirm their clinical applicability, effectiveness, and long‐term performance in peri‐implant disease prevention and management.

## 5 Conclusions

The findings of this in vitro study demonstrate that the bioadhesive gel formulated with biocidal agents significantly inhibits microbial growth and prevents the formation of three‐dimensional biofilms on dental implant surfaces after 10 days of exposure. This inhibitory effect was consistently observed across all tested surface types (sandblasted, ContacTi, and machined), highlighting the broad‐spectrum antimicrobial activity of the gel under controlled experimental conditions. However, these results should be interpreted within the limitations inherent to a preventive in vitro experimental model employing a static polymicrobial biofilm system and standardized gel application conditions that may not fully reproduce clinical scenarios. Future in vivo investigations and well‐designed clinical studies are required to confirm the clinical applicability and long‐term effectiveness of these findings in peri‐implant disease prevention and management.

## Author Contributions

María Baus‐Domínguez, José‐Luis Gutiérrez‐Pérez, and Daniel Torres‐Lagares conceived the study. María Baus‐Domínguez, Fernando Vivancos‐Cuadras, and Daniel Torres‐Lagares developed the methodology. María Baus‐Domínguez, María‐Ángeles Serrera‐Figallo, and Daniel Torres‐Lagares conducted the investigation. Paula Hermida‐Cabrera and Javier Pascual performed the formal analysis. Fernando Vivancos‐Cuadras provided resources. María Baus‐Domínguez, Paula Hermida‐Cabrera, Javier Pascual, and María‐Ángeles Serrera‐Figallo validated the data. María Baus‐Domínguez, José‐Luis Gutiérrez‐Pérez, and Daniel Torres‐Lagares supervised the project. María Baus‐Domínguez, Paula Hermida‐Cabrera, and Javier Pascual wrote the original draft. María Baus‐Domínguez, Paula Hermida‐Cabrera, Javier Pascual, Fernando Vivancos‐Cuadras, María‐Ángeles Serrera‐Figallo, José‐Luis Gutiérrez‐Pérez, and Daniel Torres‐Lagares reviewed and edited the manuscript. All authors have read and approved the final version of the manuscript.

## Ethics Statement

This study was conducted entirely in vitro using commercially available titanium implant disks and bacterial strains. No human participants, animal subjects, or clinical samples were involved. Therefore, ethical approval from an institutional review board or ethics committee was not required.

## Conflicts of Interest

Fernando Vivancos‐Cuadras is an employee of LACER S.A., the company that funded this research and manufactures the bioadhesive gel evaluated in this study. His contribution was limited to project coordination, funding management, and manuscript structuring. He was not involved in experimental design, data acquisition, microbiological analyses, or data interpretation. All experimental procedures, data analyses, and interpretation were conducted independently by the academic research team. The other authors declare no conflicts of interest.

## Data Availability

The data supporting this study's findings are available on request from the corresponding author.
